# Signal Transduction Pathways of Acupuncture for Treating Some Nervous System Diseases

**DOI:** 10.1155/2019/2909632

**Published:** 2019-07-11

**Authors:** Hsiang-Chun Lai, Qwang-Yuen Chang, Ching-Liang Hsieh

**Affiliations:** ^1^Department of Chinese Medicine, China Medical University Hospital, Taichung 40447, Taiwan; ^2^Department of Family Medicine, Lin Shin Hospital, Taichung 408, Taiwan; ^3^Chinese Medicine Research Center, China Medical University, Taichung 40402, Taiwan; ^4^Research Center for Chinese Medicine and Acupuncture, China Medical University, Taichung 40402, Taiwan; ^5^Graduate Institute of Integrated Medicine, College of Chinese Medicine, China Medical University, Taichung 40402, Taiwan

## Abstract

In this article, we review signal transduction pathways through which acupuncture treats nervous system diseases. We electronically searched the databases, including PubMed, MEDLINE, clinical Key, the Cochrane Library, and the China National Knowledge Infrastructure from their inception to December 2018 using the following MeSH headings and keywords alone or in varied combination: acupuncture, molecular, signal transduction, genetic, cerebral ischemic injury, cerebral hemorrhagic injury, stroke, epilepsy, seizure, depression, Alzheimer's disease, dementia, vascular dementia, and Parkinson's disease. Acupuncture treats nervous system diseases by increasing the brain-derived neurotrophic factor level and involves multiple signal pathways, including p38 MAPKs, Raf/MAPK/ERK 1/2, TLR4/ERK, PI3K/AKT, AC/cAMP/PKA, ASK1-JNK/p38, and downstream CREB, JNK, m-TOR, NF-*κ*B, and Bcl-2/Bax balance. Acupuncture affects synaptic plasticity, causes an increase in neurotrophic factors, and results in neuroprotection, cell proliferation, antiapoptosis, antioxidant activity, anti-inflammation, and maintenance of the blood-brain barrier.

## 1. Introduction

Acupuncture is a form of therapy practiced for more than 3000 years in Asia. Medical doctors practice acupuncture under the guidance of meridian theory to achieve “de qi” status [1]. To perform acupuncture, doctors use thin and sterile metal needles to penetrate specific stimulation points termed acupoints. Both manual and electroacupuncture (EA) are used in medical practice. Many studies have reported the benefits of acupuncture for treating diseases such as stroke, musculoskeletal disorders, chronic urticaria, irritable bowel syndrome, overactive bladder, cancer-related fatigue, and pain in humans [2–6]. Furthermore, few adverse effects have been observed when acupuncture is performed correctly, even in children and pregnant women [7, 8]. The widely known mechanism of acupuncture is that it results in the secretion of endorphins that exert an analgesic effect. With advances in understanding, more mechanisms of acupuncture have been determined, including the local segmental effect, somatoautonomic reflex, immune system regulation, neurotransmitter modulation, the neuroendocrine effect, and the functional connectivity neural network [9–11].

Nowadays, signal transduction has been applied for explaining acupuncture mechanisms. The signal transduction pathway of acupuncture has been mentioned with respect to many diseases, including neurological [12], cardiovascular [13], metabolic [14], and gynecological [15] diseases. Among the aforementioned diseases, nervous system diseases are the most common complaints in daily practice. When used to treat nervous system diseases, acupuncture enhances cell proliferation and neuroblast differentiation by increasing the levels of brain-derived neurotrophic factor (BDNF) and phosphorylated cyclic AMP response element-binding (CREB) protein [16]. Acupuncture was reported to exert a neuroprotective effect on dopaminergic neurons through anti-inflammatory and neurotrophic effects [17]. Other mechanisms, including antioxidation, antiapoptosis, and improved energy metabolism in the brain, have been reported [18–20]. Although many studies on the signal transduction pathway of acupuncture have been conducted, few reviews have been published on this topic. In the present review, we discuss the involvement of the signal transduction pathway as a mechanism underlying the effects of acupuncture when used for treating nervous system diseases.

## 2. Method

**Figure 1:**
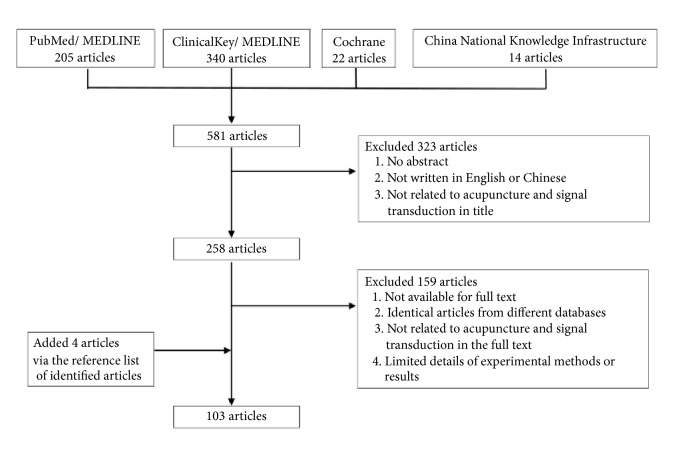
Flow chart of the search processes. The 103 articles were summarized in Tables 1–7.

We electronically searched the databases, including PubMed, MEDLINE, clinical Key, the Cochrane Library, and the China National Knowledge Infrastructure from their inception to December 2018 using the following MeSH headings and keywords alone or in varied combination: acupuncture, molecular, signal transduction, genetic, cerebral ischemic injury, cerebral hemorrhagic injury, stroke, epilepsy, seizure, depression, Alzheimer's disease (AD), dementia, vascular dementia (VD), and Parkinson's disease (PD). In addition, we used Boolean operators (“not,” “and,” ”or”) to narrow or widen search results. All articles written in English or Chinese were manually screened, and relevant studies were identified. We included additional articles after performing a manual review of the reference lists of identified studies or review articles. Excluded articles included those with unavailable full text, those written in other languages, those not mainly related to the mechanism of the signal transduction pathway, or those with limited details of experimental methods or results. Flowchart of the search processes was as shown in Figure 1.

## 3. Cerebral Ischemic Injury

Ischemic injury of the brain, also known as cerebral infarction, is a crucial health issue in the modern world because of its associated disability and socioeconomic burden. Acupuncture has shown beneficial effects on ischemic stroke rehabilitation by exerting the antiapoptosis effect on the ischemic area, promoting neurogenesis and cell proliferation, and regulating cerebral blood flow [21, 22]. A retrospective cohort study reported that acupuncture was effective at reducing the stroke recurrence rate [23]. Ischemic stroke causes neural cell damage related to excitotoxicity, oxygen free radical injury, inflammatory status, and blood-brain barrier (BBB) damage [24]. Experimental pathways that can reverse apoptosis and improve cell proliferation and differentiation have been proposed.

Acupuncture causes an increase in the expression of neurotrophic factors, such as BDNF and glial-derived neurotrophic factor (GDNF), in the central nervous system (CNS), exerts a neuroprotective effect on hypoxic-ischemic insults, and results in neurogenesis after the reconstruction phase [25, 26]. In addition, acupuncture increased the vascular endothelial growth factor (VEGF) level in the hippocampus, promoting the proliferation and differentiation of neuronal stem cells [27]. Thus, acupuncture can be used to treat ischemic injury in the brain. Zhang et al. performed manual acupuncture on GV20 and Ex-HN 1 to increase GDNF and BDNF levels in a rat model [19]. The elevation of the BDNF level was related to the increased expression of BDNF/tyrosine receptor kinase B (TrkB) and the induction of neurogenesis [28].

The mitogen-activated protein kinase (MAPK) family includes ERK1/2, JNK, and p38 MAPK proteins. In animals, the MAPK family is triggered by growth factors, stress, or an inflammatory environment and regulates cell functions, such as proliferation, division, differentiation, survival, and apoptosis. EA can trigger the MAPK family. ERK is believed to mediate reperfusion injury by inhibiting inflammatory reactions and promoting cell proliferation and growth [29]. However, equivocal results have been reported concerning the protective effect of ERK on ischemic brain injury [30, 31]. Some studies have demonstrated that EA protects against ischemic brain injury by reducing infarct volumes and improving neurological outcomes through activation of the ERK1/2 signaling pathway [29, 32–34]. EA is reported to be effective in neuroprotection and neural cell proliferation. The chosen acupoints in EA include GV20, GV14, ST36, and LI11. The activation of the ERK pathway is combined with an increase in BDNF and p-ERK1/2 levels [34]. Some studies have demonstrated that the application of EA on LU5, LI4, ST36, and SP6 was effective in reducing neurogenic deficits and causing antiapoptosis in the brain cortex and hippocampus [35, 36].

Environmental stresses and inflammatory cytokines activate p38 MAPKs and induce apoptosis and inflammation [37]. In the acute phase of ischemic brain injury, the p38 MAPK signaling pathway induces neurotoxicity, whereas in the subacute phase, this pathway serves as a proinflammatory mediator in the neuroprotective antiapoptosis effect [38–40]. Some studies have reported that EA exerts the antiapoptosis effect on the peri-infarct cortex by modulating the ERK/JNK/p38 MAPK signaling pathway [41–44]. The chosen acupoints include GV14, GV20, GV24, GV26, LU5, LILI4, LI11, ST36, and SP6. Liu et al. reported that EA inhibits microglia-mediated neuroinflammation mediated by nuclear factor kappa-light-chain-enhancer of activated B (NF-*κ*B) cells, p38 MAPK, and myeloid differentiation primary response 88 (MYD88), as well as simultaneously reducing cytokine tumor necrosis factor-alpha (TNF-*α*), interleukin-1 beta (IL-1*β*), and interleukin-6 (IL-6) levels [45].

The p38 MAPK pathway activates the expression of CREB protein and reduces the apoptosis of ischemic neural cells. Acupuncture on GV16, GV20, GV24, ST36, and HT7 also triggered the CREB pathway in the hippocampus and improved cognitive impairment in an animal model [46–51]. The CREB pathway is related to BDNF, p38 MAPK, and Ca^2+^/calmodulin-dependent protein kinase (CaMK) [46, 50, 52]. Lin et al. reported that EA exerted antioxidant and antiapoptosis effects by increasing superoxide dismutase and glutathione peroxidase levels and reducing the malondialdehyde level in the hippocampus and improved the learning and memory ability of rats [48]. A study reported that laser acupuncture on GV20 and HT7 for 14 days excited the cholinergic system and increased CREB, BDNF, and B-cell lymphoma 2 (Bcl-2) levels, thereby improving cognitive impairment in rats [51].

Being a cell cycle initiator, PI3K/AKT pathways are essential for cell survival [53]. However, interactions between transactivation of Raf/MAPK/ERK1/2 and PI3K/AKT systems were noted during ischemia and reperfusion phases. During ischemia, Akt reduces Raf/MAPK/ERK1/2 activity through phosphorylation of Raf-1. During reperfusion, abrupt reactive oxygen species (ROS) increases the phosphatase and tensin homolog and reactivates Raf/MAPK/ERK1/2 signaling [54]. For the modulation of the PI3K pathway, some studies have reported that EA on GV12, GV20, GV24, GV26, KI1, LI11, and ST36 activates the PI3K/AKT pathway and exerts antiapoptosis and neuroprotective effects [12, 55–60]. The effect of EA on the PI3K pathway can activate the downstream mTOR complex 1–UNC-51-like kinase 1 complex–Beclin-1 pathway, reduce caspase-3, caspase-8, and caspase-9 levels, and inhibit the autophagy process [61, 62]. EA also reduces nitric oxide (NO), neuronal NO synthase (nNOS), and inducible NO synthase (iNOS) levels by activating the PI3K pathway [58]. Xie et al. demonstrated that EA improved neurological deficit scores and increased the expression of p-AKT protein and bone marrow CD34+ endothelial progenitor cells in rats [63].

Because of the balance between Raf/MAPK/ERK1/2 and PI3K/AKT systems, some studies have included the pretreatment protocol [64, 65]. EA pretreatment in a rat model reduced the expression of p-Akt protein and prevented the downregulation of tight junction proteins, namely, claudin-5 and occludin, attenuating BBB disruption and brain edema [65].

NF-*κ*B is another protein complex related to cell survival. Some studies have demonstrated that EA regulates the NF-*κ*B-mediated apoptosis pathway and provides neuroprotection [66, 67].

Acupuncture improved neurogenic defects and cognitive impairment in a cerebral ischemic/reperfusion animal model. In summary, acupuncture not only increases the levels of neurotrophic factors but also modulates signaling pathways, such as Raf/MAPK/ERK1/2 and PI3K/AKT and downstream CREB and NF-*κ*B. Therefore, acupuncture results in cell proliferation, antiapoptosis, neuroprotection, and BBB maintenance. The most frequently chosen acupoints include GV20, GV14, and ST36. The mechanisms and main results of identified articles are summarized in Table 1.

## 4. Cerebral Hemorrhagic Injury

Hemorrhagic stroke is less common than ischemic stroke. The causes of hemorrhagic stroke include high blood pressure, brain trauma, aneurysms, arteriovenous malformations, and brain tumors. In cerebral hemorrhagic injury, blood vessel spasms and oxidative stress caused by ischemia and reperfusion cause an injury to neural cells. Acupuncture could improve the hypoperfusion status and hematoma absorption, reduce brain edema, and promote neurogenesis in the brain [68]. Thus, some studies have reported that acupuncture is beneficial for treating cerebral hemorrhage because it results in functional improvements [69, 70]. Acupuncture also regulates inflammatory factors, such as IL-6, IL-1*β*, and NF-*κ*B, prevents apoptosis by reducing the expression of p53 protein, and promotes neurogenesis by increasing the levels of BDNF and nerve growth factors [71].

Acupuncture increased the expression of endogenous GDNF and inhibited the early expression of VEGF, thus regulating nerve remodeling after cerebral hemorrhagic injury [72]. At the level of molecular signal transduction, acupuncture exerts a neuroprotective effect by increasing the angiopoietin level and reducing TNF-*α* and NF-*κ*B levels [73, 74]. Li et al. reported that EA on GV20 and GB7 could reduce BBB permeability and improve brain edema by activating the caveolin-1/matrix metalloproteinase pathway [75]. Antiapoptosis is also an important pathway for neural preservation. Zhu et al. and Li et al. have demonstrated that EA activated the Bcl-2 pathway to increase hematoma absorption and antiapoptosis. This effect is combined with the suppression of caspase-3 and Bcl-2-associated X (Bax) proteins [76, 77]. However, the chosen acupoints were heterogeneous, including ST36, GV14, GV20, GV26, GB7, and PC6.

Taken together, acupuncture could improve neurogenic disability and reduce brain edema by increasing caveolin-1/matrix metalloproteinase levels and inducing antiapoptosis through the activation of the Bcl-2 pathway in a cerebral hemorrhagic model. The mechanisms and main results of identified articles are summarized in Table 2.

## 5. Seizure

Seizure is an abrupt, spontaneous, excessive, or synchronous neuronal activity in the brain that leads to various uncontrolled shaking movements or loss of consciousness. Seizure attack affects 8%–10% of the general population in their lifetimes. The recurrence of seizure results in epileptic syndrome, which affects 2%–3% of the general population [78]. Epileptic seizures can be induced by metabolic imbalance, electrolyte imbalance, encephalitis, traumatic brain injury, brain tumor, stroke, and medication [78]. During the process of an epileptic seizure, changes occur in molecular, anatomical, or circuit development, including cell death, inflammatory cytokine production, and neurotransmitter dysregulation. This process is called epileptogenesis [79]. Involvement of BDNF–TrkB signaling, the mTOR pathway, and the repressor element 1-silencing transcription factor pathway was considered to be the underlying molecular mechanism [79].

In addition to the use of medication, some studies have reported that acupuncture reduced the frequency of seizures and improved the quality of life [80–82]. Some studies reported that acupuncture has effect on change of anatomical, neurotransmitter, inflammatory cytokines and molecular level. The augmentation of *γ*-aminobutyric acid neurotransmission, including the upregulation of glutamic acid decarboxylase 67 (GAD67), is a self-protective and anticonvulsive mechanism [83, 84]. Acupuncture reduced seizure attacks by enhancing GAD67 mRNA production in the dentate gyrus of epileptic rats [85]. Acupuncture changed the brain structure and reduced the mossy fiber sprouting in the dentate gyrus and exerted an antiepileptic effect [86]. Inflammation can increase neuronal excitability and result in the frequent onset of epilepsy, which is related to epileptogenesis [87]. Acupuncture also contributes to the antiepileptic effect accompanied by the anti-inflammatory effect of reducing IL-1*β*, TNF-*α*, and cyclooxygenase-2 (COX-2) levels in the hippocampus of an epileptic rat model [88, 89]. Wang et al. and Wang et al. have demonstrated that EA attenuated the seizure-induced increase in c-fos protein and preproenkephalin messenger ribonucleic acid (mRNA) levels in the hippocampus of a penicillin-induced seizure rat model [90, 91]. Yang et al. reported that EA on GV16 and GV8 exerted an anticonvulsant effect combined with a reduction in nNOS and iNOS levels [92].

With regard to molecular pathways, acupuncture on the auricular acupoint suppressed transient receptor potential ankyrin 1 (TRPA1) pathways by increasing the phosphorylated protein kinase C (pPKC)-*α* level and reducing pPKC*ε* and pERk1/2 levels in a kainic acid-induced rat model [93]. Liao et al. used a similar rat model and reported that acupuncture exerted an antiepileptic effect by inactivating the Toll-like receptor 4 (TLR4) pathway, which was accompanied by a decrease in pCaMKII*α*, pERK, pp38, pJNK, and pNF*κ*B levels [94]. Yang et al. demonstrated that acupuncture on GV20 and GV14 reduced epileptic seizures by exerting a protective effect on the pyramidal cells of hippocampal CA 1 and CA 3. This effect was related to the activation of the PI3 K/Akt pathway [95]. The upregulation of glucose-regulated protein 78 (GRP78) and the downregulation of C/EBP homologous protein (CHOP) prevent neuronal cell death induced by endoreticulum stress. Acupuncture on GV20 and GV14 elevated the GRP78 level, reduced CHOP and caspase-12 levels, and exerted an antiapoptosis effect on the hippocampus, thus reducing epileptic seizure attacks [96, 97].

Taken together, acupuncture exerts the antiepileptic effect by changing anatomical, neurotransmitter, inflammatory cytokines and molecular level. With respect to signal transduction, acupuncture reduces seizure frequency by suppressing TRPA1/pERK and TLR4/ERK pathways and activating the PI3K/Akt pathway. Furthermore, acupuncture augments the antiapoptosis process and provides neuroprotection by increasing the GRP78 level and reducing the CHOP level. The mechanisms and main results of identified articles are summarized in Table 3.

## 6. Depression

Depressive disorders are common psychiatric disorders that affect approximately 17% of people in their lifetimes. A study reported that 12%–20% of depressed patients experience treatment-resistant depression, resulting in a considerable social burden [98]. In addition to medication and psychosocial support, acupuncture serves as an alternative option for patients with depression that exhibits promising effects and fewer side effects [99]. The mechanism of depression includes dysregulation of neuroinflammatory cytokines, neurotransmitters, neuroplasticity, and the neuroendocrine system [100, 101]. At the molecular level, dysregulation of striatal-enriched tyrosine protein phosphatase inactivates the neuronal signaling pathway, including ERK1/2, p38, Src family tyrosine kinases, and glutamate receptors. This process attenuates the neurogenesis effect of BDNF and causes depression [102].

Acupuncture treats depression by regulating neurotransmitters, neuroinflammatory cytokines, the hypothalamus–pituitary–adrenal axis, and the hypothalamus–pituitary–sex gland axis [103]. Furthermore, acupuncture plays a role in molecular signaling pathways. Acupuncture elevated BDNF production and excitatory amino acid transporter levels and maintained neural regeneration of the hippocampus in a depressive rat model [104, 105]. The chosen acupoints include GV20, EX-HN3, and PC6 [104, 105]. Fan et al. demonstrated that acupuncture on LI4 and LR3 regulated the expression of soluble N-ethylmaleimide-sensitive factor attachment receptor protein, a fusion mediator, and promoted depression remission [106]. NO is a small molecule that freely diffuses across cell membranes and serves as a neurotransmitter in the CNS. NO initiates the NO-cyclic guanosine monophosphate (NO-cGMP) pathway and activates protein kinases. Acupuncture regulates the NO-cGMP pathway by increasing nNOS and cGMP levels, which contribute to its effect on depression relief [107]. Shao et al. demonstrated that acupuncture on GV20 and PC6 inhibited the proinflammatory pathway of depression by reducing NF-*κ*B protein and COX-2 levels [108].

Antidepressants alleviate the symptoms of depression by activating the MAPK/ERK pathway, which increases ERK1/2 and p-ERK1/2 expression. Many studies have reported that acupuncture activates the MAPK/ERK pathway and downstream CREB pathway and elevates BDNF production [109–114]. The most commonly chosen acupoints include GV20 and GV29, followed by EX-HN3, GB34, and PC6. The MAPK/ERK pathway induces neurogenesis and antiapoptosis of hippocampal neurons and eliminates the depression state. EA on GV20 and EX-HN3 also enhances the p-p38MAPK pathway [111]. Some studies have reported that EA on GV20 and GV29 reduced the hippocampal neural apoptotic rate by downregulating the hippocampal p-JNK pathway in depression rat model [115, 116]. Acupuncture also activated the adenyl cyclase (AC)–cyclic adenosine monophosphate (cAMP)–protein kinase A (PKA)–CREB signaling pathway and elevated the BDNF level [117–120]. In the AC–cAMP–PKA–CREB signaling pathway, heterogeneous acupoints were chosen, including GV20, EX-HN1, EX-HN3, ST36, ST40, LI4, and LR3.

Molecular studies have reported that acupuncture plays a role in the neuroendocrine model of depression. Lu et al. demonstrated that acupuncture could relieve the symptoms of depression and increase cortisol, PKA, and PKC levels [117]. Oh et al. reported that acupuncture on HT8 elevated the serum corticosterone level and hippocampal mTOR phosphorylation, Akt, ERK, p70S6K, p4E-BP1, and CREB enhanced the effect of BDNF on neuroprotection and synaptic plasticity. Furthermore, acupuncture elevated the levels of synaptic proteins (e.g., PSD95, Syn1, and GluR1), which are crucial for neuronal synaptic plasticity [121].

The results of the Gene Ontology functional term and Kyoto Encyclopedia of Genes and Genomes database analysis indicated that the regulation of the Toll-like receptor signaling pathway, nucleotide-binding oligomerization domain-like receptor signaling pathway, MAPK/ERK pathway, PI3K/Akt pathway, neurotrophin signaling pathway, TNF pathway, and NF-*κ*B pathway is the mechanism through which acupuncture treats depression. The aforementioned pathways cause cell survival, differentiation, antiapoptosis, and synaptic plasticity of neurons, thus alleviating depression symptoms and improving learning/memory dysfunction [122–124].

In summary, acupuncture can treat depression by upregulating MAPK/ERK and AC–cAMP–PKA–CREB pathways and downregulating JNK and NF-*κ*B pathways. Because of the aforementioned mechanism, we observed an increase in neuron growth factor levels, neurogenesis, and antiapoptosis accompanied by the alleviation of depression symptoms. The mechanisms and main results of identified articles are summarized in Table 4.

## 7. Alzheimer's Disease

AD is a progressive neurodegenerative disease that is presented with dementia, memory loss, disorientation, personality disorder, mood swings, behavior disturbance, and language problems. Because of patients' cognitive decline, they withdraw from their family and society [125]. Risk factors for AD include genetic factors, a history of head trauma, depression, and hypertension [126]. The progression of AD is associated with the formation of amyloid plaques and neurofibrillary tangles in the brain [126]. Treatment of AD should be started immediately after the diagnosis to prevent cognitive decline. Both patients and their families are involved in administration of medication and psychosocial therapy for AD. Medication for AD includes cholinesterase inhibitors (donepezil, rivastigmine, and galantamine), N-methyl-D-aspartate receptor antagonists (memantine), atypical antipsychotics, antidepressants, and anticonvulsants [126].

In addition to medication, acupuncture has been reported to improve cognitive function and the global clinical status of patients with AD without causing major adverse effects [127, 128]. Mechanisms through which acupuncture improves cognitive impairment in AD include attenuation of A*β* deposits, upregulation of BDNF expression, and regulation of cell proliferation and neural plasticity in the brain [129–131]. Acupuncture also regulates cytokine and growth factor levels associated with survival, proliferation, and differentiation of neural stem cells in the brain to promote the repair of damaged cells [130, 132].

A*β* deposits in the brain disturb BDNF signaling pathways, such as Ras/ERK, PI3K/Akt, and PKA/cAMP, which regulate BDNF expression and cause AD development [133, 134]. Acupuncture on GV20 reduces A*β* deposits in the brain, elevates the BDNF level, and exerts a neuroprotective effect on CNS cells [135, 136]. Lin et al. reported that the signaling pathway of BDNF elevation is mediated by the BDNF–TrkB pathway, which exerts an antiapoptosis effect [136]. The central cholinergic pathway is important for learning acquisition and synaptic plasticity in the mammalian limbic system; thus, increasing the acetylcholine level is a type of treatment strategy for AD. Lee et al. reported that acupuncture enhances the cholinergic system–CREB–BDNF pathway and exerts a neuroprotective effect [135].

The p38 MAPKs are activated by environmental stresses and inflammatory cytokines and induce apoptosis and inflammation. In an AD animal model, acupuncture could improve cognitive impairment by reducing p38 MAPK levels, thus reducing neuroinflammation in the CNS [18, 137, 138]. Some studies have reported using Sanjiao acupuncture, which uses CV17, CV12, CV6, ST36, and SP10, as a standard regimen for AD [18, 139, 140]. A DNA microarray analysis demonstrated that Sanjiao acupuncture could reverse gene expression profiles related to aging in the hippocampus of senescence-accelerated mouse prone 10 (SAMP10) mice and reduce oxidative stress–induced damage [18]. Luo et al. reported that Sanjiao acupuncture attenuated cognitive deficits by regulating the G-protein/inositol triphosphate/Ca^2+^ amplitude pathway and signal homeostasis [140]. In an A*β*-induced AD model, acupuncture on GV20 and BL23 reduced the level of peroxisome proliferator-activated receptor-*γ* (PPAR-*γ*) level and the deposition of Tau protein, thus reducing neuroinflammation [138].

Acupuncture regulated cell cycle and aging in an AD model. N-myc downregulated gene 2 (NDRG2) encodes a cytoplasmic protein that may play a role in neurite outgrowth. Wang et al. demonstrated that EA on GV20 suppressed the astrocyte NDRG2 expression and glial fibrillary acidic protein level, thereby treating memory impairment of amyloid precursor protein/presenilin-1 double transgenic mice [141]. P130, known as retinoblastoma-like protein 2 (RBL2), is a protein encoded by the* RBL2* gene in humans and serves as a tumor suppressor signal. Acupuncture on CV17, CV12, CV6, SP10, and ST36 elevated the p130 level, caused cell proliferation in the brain, and treated dementia and aging-related diseases in SAMP10 mice [139]. Telomerase is a critical enzyme involved in aging and apoptosis. Lin et al. demonstrated that acupuncture on ST35 of telomerase-deficient mice activated the BDNF–TrkB signaling pathway along with elevating BDNF, TrkB, Akt, and ERK1/2 levels, which resulted in an increase in telomerase activity [142]. Acupuncture also modulates the balance of Bcl-2/Bax to regulate the cell cycle of neurons. However, the chosen acupoints were heterogeneous, including LI20, EX-HN3, GV20, BL23, and KI1 [143–145].

Metabolic stress modulates *β*-secretase gene transcription and *β*-site amyloid precursor protein-cleaving enzyme 1 (BACE1) protein levels in AD through the sirtuin 1 (SIRT1)-PPAR*γ*-proliferator-activated receptor *γ* coactivator 1 (PGC-1) pathway [146]. A*β* 25–35 suppresses mitochondrial biogenesis by inactivating the AMP-activated protein kinase (AMPK)–SIRT1–PGC-1*α* pathway in hippocampal neurons [147]. Therefore, brain energy metabolism impairment is considered an underlying pathogenesis of AD progression. Acupuncture on GV20 elevates glucose transporter (GLUT1 and GLUT3), p-AMPK, p-AKT, and mTOR levels in the hippocampus and cortex. Through regulation of brain energy metabolism, acupuncture has effect on decreasing A*β* deposits, suppressing autophagy process and relieving cognition deficits [148]. Acupuncture improved the spatial learning and memory ability of AD mice by increasing blood perfusion and glucose uptake in the bilateral amygdala, hippocampus, and left temporal lobe [149, 150]. For the molecular signaling pathway, Dong et al. demonstrated in two series studies that acupuncture in GV14 and BL23 exerted AMPK expression, activated SIRT1-PPAR*γ*- PGC-1 pathway, and elevated ATP level. Because of the aforementioned mechanism, acupuncture balances brain metabolism and improves cognition impairment of AD mice [20, 151]. Furthermore, the upregulation of SIRT1–PPAR*γ*–PGC-1 suppresses BACE1 expression, thus reducing A*β* production in the hippocampus and improving cognitive decline in SAMP8 mice [152].

In summary, acupuncture treats AD by regulating neurotransmitter release, elevating the neurotrophic factor level, and exerting anti-inflammatory effects. Thus, many molecular signaling pathways involved in acupuncture were reported in the AD model, including the BDNF–TrkB pathway, the cholinergic system–CREB–BDNF pathway, G-protein regulation, and the p38 MAPK family. The aforementioned pathways are believed to exert antiapoptosis and anti-inflammatory effects and reduce A*β* deposits in the brain, thereby improving learning ability and memory in AD models. The most commonly chosen acupoints were GV20 and the Sanjiao regimen (CV17, CV12, CV6, ST36, and SP10). Acupuncture regulates cell cycle and aging by modulating NDRG2 and P130 expression, telomerase activity, and Bcl-2/Bax balance. Many studies have reported that acupuncture on GV14 and BL23 modulates brain energy metabolism impairment and treats cognitive impairment. The mechanisms and main results of identified articles are summarized in Table 5.

## 8. Vascular Dementia

VD, which accounts for 15% of dementia cases, is the second most common cause of dementia after AD. Multiple and recurrent ischemia of the brain caused by ischemia or hemorrhage has been found to be the main causes of VD [169]. Although the pathophysiology of VD remains unclear, approximately 15%–30% of patients develop dementia three months after the occurrence of stroke. Furthermore, approximately 20%–25% of patients develop delayed dementia [170]. Because of intricate coordination in the brain and, sometimes, the presence of other brain damage causes, the cognitive changes and declines in VD can be variable, including impairment of attention, information processing, and executive function [169]. Few medications have been approved specifically for the prevention or treatment of VD. Thus, treatment strategies for VD are similar to those for AD and include the use of cholinesterase inhibitors and memantine and providing psychosocial support.

Acupuncture can improve the scores on the Mini-Mental Status Examination, the revised Hasegawa's dementia scale, and activities of daily living examination for VD patients [171, 172]. From the molecular viewpoint, acupuncture on GV20 and KI3 regulates the MAPK/ERK pathway by elevating the pERK level and reducing ionized calcium-binding adaptor molecule 1 (Iba-1), TLR4, and TNF-*α* levels [153]. Acupuncture reduced relevant proinflammatory factors, thus attenuating neuroinflammation and increasing neuronal synaptic plasticity.

Acupuncture exerted antioxidant and antiapoptosis effects in VD models. Zhu et al. reported that acupuncture on GV20 and ST36 inactivated the apoptosis signal-regulating kinase 1 (ASK1)–JNK/p38 pathway and elevated thioredoxin-1 and thioredoxin reductase-1 levels [154]. The p38 MAPK pathway activates the expression of CREB and reduces the apoptosis of ischemic neural cells. Some studies have reported that acupuncture activates the cAMP/PKA/CREB pathway and elevates the CREB level [47, 48, 50, 51]. The elevated CREB level upregulates Bcl-2 activity and downregulates Bcl-2xl and Bax activities, consequently preventing the apoptosis of neurons injured by vascular events [48, 51]. The most discussed acupoint was GV20, followed by GV24. Scalp and Sanjiao acupuncture techniques (CV17, CV12, CV6, ST36, and SP10) have been reported to affect the balance between Bcl-2 and Bax expression and antiapoptosis [155, 156]. VD rats had lower expression of mTOR and eukaryotic translation initiation factor 4E (eIF4E) in CA1 accompanied with decreased spatial memory [173]. Zhu et al. demonstrated that EA on GV20, GV14, and BL23 activates the mTOR pathway and increases mTOR and eIF4E levels, thus modulating cell growth, proliferation, and synaptic plasticity [157].

Taken together, acupuncture treats VD by activating MAPK/ERK and ASK1–JNK/p38 pathways; increasing CREB, mTOR, and Bcl-2 levels; and reducing the Bax level. In addition, through the aforementioned mechanism, acupuncture exerts an effect on antioxidant activity, antiapoptosis, and synaptic plasticity. The most commonly chosen acupoints were GV20, GV24, and ST36. The mechanisms and main results of identified articles are summarized in Table 6.

## 9. Parkinson's Disease

PD is a chronic neural degenerative disorder that mainly affects the motor system. Patients with PD experience shaking, rigidity, and walking difficulty. In advanced stages of the disease, behavioral disturbance, depression, poor sleep, and cognitive dysfunction are noted [174]. Treatments such as the administration of L-dopa, dopamine agonists, catechol-O-methyl transferase inhibitors, and monoamine oxidase inhibitor and deep brain stimulation are suggested for treating motor problems of patients with PD. However, dyskinesias and motor fluctuations that develop after a long-term use or high dose use of L-dopa and nonmovement-related symptoms, such as sleep disturbances and psychiatric problems, become problems for patients with PD [174].

Both manual acupuncture and EA help alleviate some motor symptoms in patients with PD and some nonmotor symptoms, such as psychiatric disorders, sleep disorders, and gastrointestinal symptoms. Acupuncture also improved the therapeutic efficacy of levodopa, lowering the necessary dosage [175–177]. Reducing dopaminergic neurons in the substantia nigra (SN) results in PD. Acupuncture has been reported to exert neuroprotective effects that increase the levels of endogenous neurotrophins and modulate the apoptosis and neuroinflammation of dopaminergic neurons in the SN [178, 179]. Neuroimaging findings of the human brain showed that acupuncture on GB34 and the scalp significantly increased glucose metabolism bilaterally in the frontal and occipital lobes and improved motor dysfunction in patients with PD [179, 180].

In light of signal transduction, EA at 2 Hz on GV16 and LR3 inactivate the ERK 1/2 signaling pathway and p38/MAPK signaling pathway, causing an increase in tyrosine hydroxylase–positive neurons and a decrease in COX-2, TNF-*α*, and IL-1*β* levels. The regulation of cytokines reduces the neuroinflammation of the SN and alleviates PD symptoms [158, 159]. Acupuncture also activates the PI3K/Akt pathway, which elevates the Bcl-2 level and reduces dopamine- and cAMP-regulated phosphoprotein of 32 kDa and Fos B. Through the activation of the PI3K/Akt pathway, acupuncture increases the dopamine turnover rate and availability in the synapse of the SN and striatum and regulates the tyrosine hydroxylase–positive cell cycle, thus improving motor function [160–162]. Lu et al. demonstrated that EA on KI3 inactivates pPKA/pPKC/CaMKII*α* signaling pathways and reduces neuronal excitotoxicity in the hippocampus [163].

Rapamycin, an inhibitor of mTOR, is a potent inducer of autophagy and has an effect on PD [181]. However, rapamycin-based treatments for PD show adverse effects, including dyslipidemia, proliferative dysregulation, and renal dysfunction [182]. Acupuncture on GB34 affected the downstream autophagy–lysosome pathway through the m-TOR-independent pathway; this effect was comparable to that observed in the rapamycin treatment group [164]. Acupuncture induced autophagic clearance of *α*-syn, caused recovery of DA neurons in the SN, and improved motor function of an animal model without any notable adverse effect [164].

Oxidative stress and inflammation both contribute to the neural toxicity and development of PD [183]. Many studies have indicated the use of high-frequency EA for treating PD motor symptoms in animal models [184, 185]. Kim et al. reported that high-frequency EA on GB34 and GB39 increased tyrosine hydroxylase–positive neurons and cytochrome c oxidase subunit Vb and reduced cytosolic malate dehydrogenase, munc18-1, and hydroxyacylglutathione hydrolase levels, thus exerting an antioxidative effect on the SN [165]. Lv et al. demonstrated that EA at 100 Hz on ST36 and SP6 exerted a neuroprotective effect on PD mice and reversed the increase in the levels of Iba-1 and proinflammatory cytokines, including TNF-*α*, IL-6, and IL-1*β*, induced by 1-methyl-4-phenyl-1,2,3,6-tetrahydropyridine (MPTP), thus suppressing the neuroinflammatory process [166]. The nuclear factor erythroid 2 -related factor 2 (Nrf2)–antioxidant response element (ARE) pathway regulates oxidative stress and inflammatory responses. EA enhances the Nrf2–ARE pathway and regulates the expression of antioxidants, such as the ARE-driven reporter gene, nicotinamide adenine dinucleotide phosphate quinone oxidoreductase, and heme oxygenase-1 (HO-1), thus relieving PD symptoms [166]. Similarly, Deng et al. reported that EA at 100 Hz on ST36 and SP6 elevated HO-1 and glutamate–cysteine ligase modifier subunits and reduced astrogliosis and neuroinflammation through the Nrf2–ARE pathway [167].

PD symptoms were relieved through the modification of TLR/NF-*κ*B and Nrf2/HO-1 pathways [186]. EA on GV16 and LR3 upregulated NF*κ*B protein expression and downregulated 26S proteasome protein expression in rotenone-induced PD rats [187]. P53 plays a role in DNA repair or cell death depending on the nature and extent of stress and damage [188]. P53 dysfunction was reported in neurodegenerative diseases and cancers [189]. Park et al. demonstrated that acupuncture on GB34 activated the p53 signaling pathway, protected dopaminergic neurons in the SN and striatum, and treated PD symptoms [168].

At the gene level, Choi et al. demonstrated that EA regulated gene expression in the striatum and exerted a neuroprotective effect on MPTP parkinsonism mice [190, 191]. Yeo et al. performed a microarray analysis study of acupuncture on GB34 and LR3 in an MPTP mouse model of parkinsonism and reported that acupuncture reversed the downregulation of five annotated genes and upregulation of three annotated genes through MPTP intoxication [192].

In summary, acupuncture improved motor dysfunction and memory of PD. These effects were accompanied by the regulation of gene expression. Acupuncture modulates neuroinflammation by inactivating ERK 1/2 and p38/MAPK signaling pathway and reduces neuronal excitotoxicity through the pPKA/pPKC/CaMKII*α* signaling pathway. Acupuncture also regulates apoptosis by balancing the Bcl-2 and m-TOR-independent pathway. The most chosen acupoints include GB34, LR3, and GV16. Moreover, high-frequency EA (100 Hz) on ST36 and SP6 reduces neuroinflammation through the Nrf2–ARE pathway. The mechanisms and main results of identified articles are summarized in Table 7.

## 10. Conclusion

**Figure 2:**
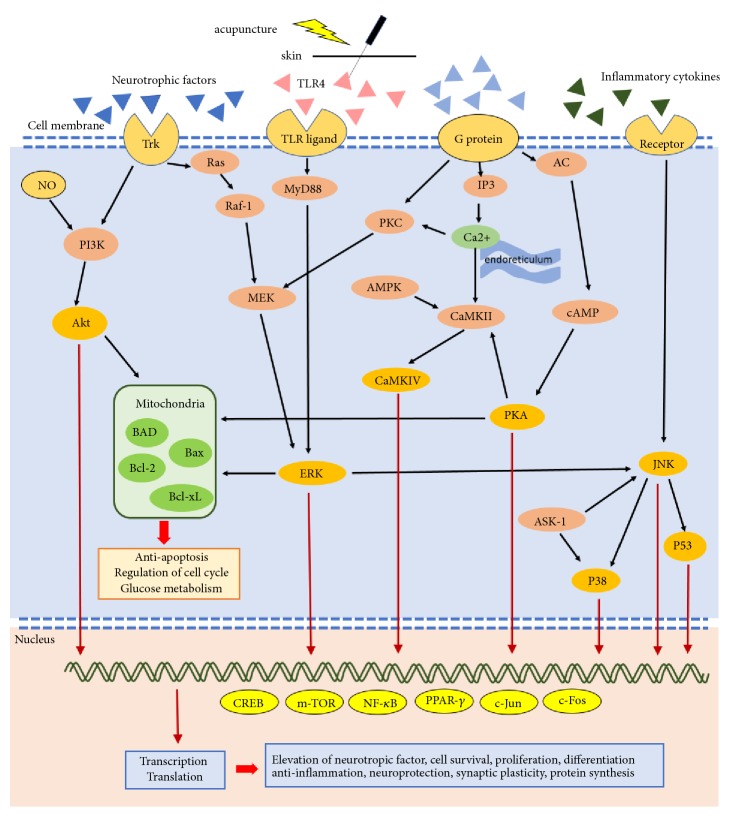
Summary of signal transduction pathways through which acupuncture treats nervous system diseases. Acupuncture is applied on acupoints and results in de qi, evoking excitation of cell membrane receptors, such as the Trk and TLR/ligand, and subsequently producing signal transduction. AC: adenyl cyclase; Akt: protein kinase B; AMPK: AMP-activated protein kinase; ASK-1: apoptosis signal-regulating kinase 1; Bad: Bcl-2-associated death promoter; Bax: Bcl-2 associated X; Bcl-2: B-cell lymphoma 2; Bcl2-xl: B-cell lymphoma-extralarge; CaMK: Ca2+/calmodulin-dependent protein kinase; cAMP: cyclic adenosine monophosphate; CREB: phosphorylated cyclic AMP response element-binding protein; ERK: extracellular signal-regulated kinase; IP3: inositol triphosphate; JNK: c-Jun N-terminal kinases; MEK: mitogen-activated protein kinase/extracellular signal-regulated kinase kinase; mTOR: mammalian target of rapamycin; MyD88: myeloid differentiation primary response 88; NF-*κ*B: nuclear factor kappa-light-chain-enhancer of activated B cells; NO: nitric oxide; PI3K: phosphatidylinositol-4,5-bisphosphate 3-kinase; PKA: protein kinase A; PKC: protein kinase C; PPAR-*γ*: peroxisome proliferator-activated receptor *γ*; TLR: Toll-like receptor; Trk: tyrosine receptor kinase.

Acupuncture treats nervous system diseases through many signal transduction pathways. Besides increasing the neurotrophic factors level, acupuncture influences pathways including p38 MAPKs, Raf/MAPK/ERK1/2, TLR4/ERK, PI3K/AKT, AC/cAMP/PKA, ASK1–JNK/p38, and downstream CREB, JNK, m-TOR, NF-*κ*B, and Bcl-2/Bax balance. We summarized the common signal transduction pathways through which acupuncture treats nervous system diseases (Figure 2). Through the aforementioned pathways, acupuncture affects synaptic plasticity, elevates neurotrophic factors, and results in neuroprotection, cell proliferation, antiapoptosis, antioxidant activity, anti-inflammation, and maintenance of the BBB.

## Figures and Tables

**Table 1:** Signal transduction pathways of acupuncture in treating cerebral ischemic injury.

Subjects	Location	Acupoint	Intervention	Time of intervention	Signal pathway	Main results	Author, reference
Male, SD rats, MCAO	brain	GV20	EA, 3mA, 2/20Hz	30min, QOD for 14 days	increase expression of BDNF/TrkB	elevation of BDNF neuron proliferation	Kim MW, et al. 2012[28]

Male, postnatal SD rats, MCAO	hippocampus	GV20, GV14	EA, 2Hz	20min, QD for 10 days	increase VEGF and BDNF levels	proliferation and differentiation of neuronal stem cells	Kim YR, et al. 2014[27]

Male, postnatal SD rats, CCAO	hippocampus	GV20, Ex-HN 1	MA, 2Hz for 15 sec	30min/time, 3 times	increase GDNF and BDNF levels	antiapoptosis	Zhang Y, et al. 2015[19]

Either sex, SD rats, CCAO combination with hypoxic treatment	cerebral cortex	MA: GV 20, GV 14, LI 11, KI 1 EA: GV 14, LI 11	MA and EA, 1mA, 1/20 Hz	10 min, QD	activation of GDNF/RET/Akt pathway	neuroprotection	Xu T, et al. 2016[25]

Male, SD rats, MCAO	brain	GV20	EA, 1 mA, 2/15 Hz	30min	activation of ERK1/2 pathway	elevation of CB1 neuroprotection	Du J et al. 2010[32]

Male, SD rats, MCAO	brain	ST36, LI11	EA, 1/20 Hz	30 min, QD	activation of the ERK pathway	elevation of Ras, cyclin D1 and CDK4 neural cell proliferation	Xie G, et al. 2013[33]

Male, SD rats, MCAO	brain	GV 20, GV14	EA,2.7-3.0 mA, 5Hz	25min, QD for 2 days	activation of MAPK/ERK kinase, ERK1/2 pathway	elevation of BDNF, pRaf-1, pp90RSK, pBad depression of caspase-3 protein neuroprotection	Cheng CY, et al. 2014[34]

Male, SD rats, MCAO	brain	LI11, ST36	EA, 1-20 Hz	30min, QD for 3 days	activation of the ERK1/2 pathway	elevation of p21 or p27 depression of cyclin D1, CDK4, cyclin E and CDK2 neural cell proliferation	Huang J, et al. 2014[29]

Male, SD rats, MCAO	hippocampus	LU5, LI4, ST36, SP6	EA, 2mA, 2/15 Hz	20 min, QD for 3 days	activation of the ERK pathway	antiapoptosis	Wu C, et al. 2015[35]

Male, SD rats, MCAO	brain	LU5, LI4, ST36, SP6	EA,2 mA, 2/15Hz	20min, QD for 3 days and 7 days	activation of ERK pathway	antiapoptosis	Wu C, et al. 2017[36]

Male, SD rats, ligation of common carotid artery and external carotid artery	hippocampus	LU5, LI4, ST36, SP6	EA, 2/50 Hz	20 min, QD	regulation of p38 MAPK signal pathway	depression of phosphorylated p38 MAPK antiapoptosis	Lan X, et al. 2017[41]

Male, SD rats, MCAO	brain	GV20, GV14, GV26	MA	30min/time, 7 times	Inactivation of MAPK/ERK pathway	elevation of Bcl-2 depression of Bax anti-apoptosis	Lin Y, et al. 2017[42]

Male, SD rats, MCAO	brain	LI11, ST36	EA, 1mA, 4/20 Hz	30min, QD for 3 days	modulation of ERK/JNK/p38 signal pathway	elevation of caspase-3, growth factor midkine depression of Bcl-2 anti-apoptosis	Xing Y, et al. 2018[43]

Male, SD rats, MCAO	brain	GV20, GV24	EA, 1mA, 1/20 Hz	30 min, QD for 10 days	modulation of p38MAPK/ERK1/2/JNK pathway	elevation of ERK1/2, Bcl-2/Bax ratio depression of JNK, p38 MAPK anti-apoptosis	Liu J, et al. 2018[44]

Male, SD rats, MCAO	sensorimotor cortex	LI11, ST36	EA, 0.2mA, 1/20Hz	30 min, QD for 3 days	inactivation of NF-*κ*B, p38 MAPK and MYD88 pathway	depression of TNF-*α*, IL-1*β*, IL-6 inhibition of microglia-mediated neuroinflammation	Liu W, et al. 2016[45]

Male, SD rats, MCAO	brain	GV20, GV16	EA, 5 Hz and 25Hz	25 min, QD	activation of p38 MAPK/CREB pathway	decrease reactive astrocytosis	Cheng CY, et al. 2015[46]

Male, Wistar rats, homologous blood emboli injection of internal carotid artery	hippocampus	ST36	MA	QD for 14 days	activation of cAMP/PKA/CREB pathway	activation of long-term potentiation	Li QQ, et al. 2015[47]

Male, SD rats, MCAO	hippocampus	GV24, GV20	EA, 1-3mA, 5/20Hz	30min, QD	increase expression of p-CREB	elevation of superoxide dismutase and glutathione peroxidase, Bcl-2 depression of malondialdehyde, Bcl2-xl anti-oxidase anti-apoptosis	Lin R, et al. 2015[48]

Male C57BL/6 mice, bilateral stenosis of the common carotid artery	corpus callosum	GV20, GV14	EA, 2Hz	20min, QD for 7 days	p-CREB pathway	oligodendrocyte regeneration	Ahn SM, et al. 2016[49]

Female, SD rats, MCAO	hippocampus	GV20, GV24	EA, 1/20Hz	30min, QD for 7 days	inactivation of CaM-CaMKIV-CREB pathway	inactivation of CaM-CaMKIV-CREB pathway	Zhang Y, et al. 2016[50]

Male, SD rats, MCAO	hippocampus	GV20, HT7	MA LA, 30 mW, 100Hz	14 days	enhance cholinergic system	elevation of CREB, BDNF, and Bcl-2 depression of Bax anti-apoptosis	Yun YC, et al. 2017[51]

Neonatal SD rats, CCAO	brain	GV20, ST36	EA, 1mA, 2Hz	20min	activation of CREB/BDNF pathway	oligodendrogenesis	Pak ME, et al. 2018[52]

Male, Wistar rats, MCAO	forebrain	GV20, GV26	EA, 3mA, 3/20Hz	60min	activation of Akt	depression of caspase-9 anti-apoptosis	Wang SJ, et al. 2002[55]

Male, SD rats, MCAO	brain	GV26, CV 24,	EA,1 mA, 4/16Hz	30min	activation of PI3K pathway	neuroprotection	Sun N, et al. 2005[56]

Male, SD rats, MCAO	brain	GV26, CV24	EA, 4/16Hz	30 min	activation of TrkA-PI3K pathway	neuroprotection	Zhao L, et al. 2007[57]

Rats, modified intravascular suture technique	hippocampus, cerebral cortex	GV26, CV 24	acupuncture	-* *-	activation of TrkA/PI3K pathway	depression of NO, nNOS and iNOS	Chen SX, et al. 2011[58]

Male, SD rats, MCAO	brain	LI11, ST36	EA, 1mA, 1/20 Hz	30 min, QD	activation of PI3K/Akt pathway	elevation of BDNF, GDNF, Bcl-2/Bax ratio anti-apoptosis	Chen A, et al. 2012[59]

SD rats, left common carotid artery (LCCA) ligation	cerebral cortex	GV 20, GV 14, LI 11, KI 1	MA and EA	-* *-	activation of PI3K/Akt pathway	neuroprotection	Xu T, et al. 2014[60]

Male, SD rats, MCAO	brain	LI11, ST36	EA, 4/20 Hz	30 min, QD for 3 days	activation of PI3K/Akt pathway	elevation of PI3K, p-Akt, p-Bad and Bcl-2 depression of Bax, caspase-3-positive expression anti-apoptosis	Xue X, et al. 2014[12]

Male, SD rats, MCAO	brain	GV20, CV6	EA,1mA, 2Hz	30min, BID	activation of PI3K/Akt pathway	depression of caspase-3, -8 and -9 anti-apoptosis	Kim YR, et al. 2013[61]

Male, SD rats, MCAO	bone marrow	GV20, LI4, LR3	EA, 3mA, 2/20Hz	30min, QD	increase expression of p-Akt protein	elevation of CD 34+ endothelial progenitor cell	Xie CC, et al. 2014[63]

Male, SD rats, MCAO	brain	LI11, ST36	EA, 0.2 mA, 1/20 Hz	30 min, QD for 3 days	activation of mTORC1-ULK1 complex-beclin1 pathway	depression of microtubule-associated protein 1 light chain 3 beta II/I, ULK1, autophagy related gene 13 and Beclin1 anti-autophagy	Liu W, et al. 2016[62]

Male, SD rats, MCAO	brain	GV20	EA, 1mA, 2/15 Hz	30 min, QD for 3 days	phosphorylation of GSK-3*β*	anti-apoptosis	Wei H, et al. 2014[64]

Male, SD rats, MCAO	brain	GV20	EA, 1mA, 2/15Hz	30min, QD for 5 days	decrease expression of p-Akt	elevation of claudin-5, occludin decrease blood-brain barrier disruption	Zou R, et al. 2015[65]

Male, SD rats, MCAO	brain	GV20, GV24	EA 1/20Hz	30min, QD for 10 days	inhibition of NF-*κ*B-mediated apoptosis pathway	depression of Bax and Fas anti-apoptosis	Feng X, et al. 2013[66]

Male, SD rats, MCAO	brain	LI11, ST36	EA,0.01mA, 1/20Hz	-* *-	regulation of TLR4/NF-*κ*B pathway	depression of TNF-*α*, IL-1*β* and IL-6 neuroprotection	Lan L, et al. 2013[67]

Abbreviations

-* *-: not mentioned; Bax: Bcl-2 associated X; Bad: Bcl-2-associated death promoter; Bcl-2: B-cell lymphoma 2; BDNF: brain-derived neurotrophic factor; CaMK: Ca2+/calmodulin-dependent protein kinase; cAMP: cyclic adenosine monophosphate; CB1: cannabinoid receptor type 1; CCAO: occlusion of common carotid artery; CDK: cyclin-dependent kinase; CREB: phosphorylated cyclic AMP response element-binding protein; EA: electroacupuncture; ERK: extracellular signal-regulated kinase; GDNF: glial-derived neurotrophic factor; IL: interleukin; JNK: c-Jun N-terminal kinases; MA: manual acupuncture; MAPK: mitogen-activated protein kinases; MCAO: occlusion of MCA; mTOR: mammalian target of rapamycin; MYD88: myeloid differentiation primary response 88; NF-*κ*B: nuclear factor kappa-light-chain-enhancer of activated B cells; p38 MAPKs: p38 mitogen-activated protein kinases; PI3K: phosphatidylinositol-4,5-bisphosphate 3-kinase; PKA: protein kinase A; pp90RSK: phospho-90 kDa ribosomal S6 kinase; QD: daily; QOD: every other day; SD rat: Sprague Dawley rat; TLR4: Toll-like receptor 4; TNF-*α*: tumor necrosis factor-alpha; Trk: tyrosine receptor kinase; ULK: UNC-51-like kinase; VEGF: vascular endothelial growth factor.

**Table 2:** Signal transduction pathways of acupuncture in treating cerebral hemorrhagic injury.

Subjects	Location	Acupoint	Intervention	Time of intervention	Signal pathway	Main results	Author, reference
Male, Wistar rats	brain	GV20, GB7	MA	30min, QD for 1,2,3,7,10 days	increase GDNF level and modulate VEGF level	elevation of GDNF, VEGF (early) depression of VEGF (late) modulate neuron remodeling	Zhang GW, et al. 2012[72]

Male, SD rats, collagenase-induced ICH	right globus pallidus	ST36	EA,2-20Hz	30min, QD, 14 days	activation of Ang-1 and Ang-2	elevation of Ang-1 and Ang-2 neuroprotection	Zhou HJ, et al. 2014[73]

Male, SD rats, autologous blood-induced ICH	right caudate nucleus	GV20, GB7	MA, 3-4Hz, 5min	30min, QD, 7 days	inactivation of TNF pathway	depression of TNF-*α* and NF-*κ*B anti-inflammation	Liu H, et al. 2017[74]

Male, SD rats, collagenase-induced ICH	right caudate nucleus	GV20, GB7	EA,0.2mA, 2Hz	30min, QD, 1,3,7 days	activation of caveolin-1/matrix metalloproteinase/blood-brain barrier permeability pathway	elevation of caveolin-1, matrix metalloproteinase-2/9 reduce blood-brain barrier permeability	Li HQ, et al. 2016[75]

Male, SD rats, collagenase and heparin-induced ICH	right caudate putamen	GV20, GV14	EA,1mA, 3Hz	10min, QD, 14 days	activation of Bcl-2 pathway	elevation of Bcl-2 protein depression of caspase-3 and Bax proteins increase absorption of hematoma and anti-apoptosis	Zhu Y, et al. 2017[76]

Male, Wistar rats, autologous blood-induced ICH	caudate nucleus	PC6, GV26	EA, 4Hz	1min	balance of BCL-2 and Bax	elevation of BCL-2 mRNA depression of Bax mRNA anti-apoptosis	Li Z, et al. 2017[77]

Abbreviations

-* *-: not mentioned; Ang: Angiopoietin; Bax: Bcl-2 associated X; Bcl-2: B-cell lymphoma 2; EA: electroacupuncture; GDNF: glial-derived neurotrophic factor; ICH: intracranial hemorrhage; MA: manual acupuncture; NF-*κ*B: nuclear factor kappa-light-chain-enhancer of activated B cells; QD: daily; SD rat: Sprague Dawley rat; TNF-*α*: tumor necrosis factor-alpha; VEGF: vascular endothelial growth factor.

**Table 3:** Signal transduction pathways of acupuncture in treating seizure.

Subjects	Location	Acupoint	Intervention	Time of intervention	Signal pathway	Main results	Author, reference
Male, SD rats, lithium-pilocarpine injection	dentate gyrus	ST36	EA, 1-20mA, 4/20Hz	30min, QD for 30,45,60 days	activation of GAD 67	elevation of GAD67 mRNA anti-epileptic	Guo J, et al. 2008[85]

Male, SD rats, kainic acid injection	prefrontal cortex, hippocampus, and somatosensory cortex	auricular acupoint	Auricular EA, 2 and 15Hz	20min, QD, 3 days/wk for 3 wks	inactivation of TLR 4 pathway	depression of pCaMKII*α*, pERK, pp38, pJNK, pNF*κ*B anti-epileptic	Liao ET, et al. 2018[94]

Male, SD rats, intraperitoneal injection of pentylenetetrazol	hippocampal CA 1 and CA 3	GV20, GV14	MA	QD for 5 days	activation of PI3 K/Akt pathway	increase pyramidal cells	Yang, F, et al. 2013[95]

Male, SD rats, kainic acid injection	hippocampal CA1 areas	Auricular acupoint	EA, 2Hz	20min, 3 days/wk for 6wks	Inactivation of TRPA1, pPKC*α*, pPKC*ε*, and pERk1/2 pathways	elevation of PKC*α* depression of TRPA1, PKC*ε*, pERK1/2 anti-epileptic	Lin YW, et al. 2014[93]

Male, SD rats, intraperitoneal injection of pentylenetetrazol	hippocampal CA 1 region	GV20, GV14	MA	30min	balance of GRP78 and CHOP	elevation of GRP 78 protein depression of CHOP neuroprotection	Yang F, et al. 2014[96]

Male, newly-born SD rats, pentylenetetrazol intraperitoneal injection	hippocampus	GV20, GV14	MA	QD for 7 days	balance of GRP78 and CHOP	elevation of GRP 78 protein depression of CHOP, caspase-12 anti-apoptosis	Zhang, H, et al. 2017[97]

Abbreviations

Akt: protein kinase B; CaMK: Ca2+/calmodulin-dependent protein kinase; CHOP: C/-EBP homologous protein; COX: cyclooxygenase; EA: electroacupuncture; ERK: extracellular signal-regulated kinase; GAD67: glutamic acid decarboxylase 67; GRP78: glucose-regulated protein 78; IL: interleukin; JNK: c-Jun N-terminal kinases; MA: manual acupuncture; NF-*κ*B: nuclear factor kappa-light-chain-enhancer of activated B cells; p38 MAPKs: p38 mitogen-activated protein kinases; PI3K: phosphatidylinositol-4,5-bisphosphate 3-kinase; PKC: protein kinase C; QD: daily; QOD: every other day; SD rat: Sprague Dawley rat; TLR4: Toll-like receptor 4; TNF-*α*: tumor necrosis factor-alpha; TRPA: transient receptor potential ankyrin 1.

**Table 4:** Signal transduction pathways of acupuncture in treating depression.

Subjects	Location	Acupoint	Intervention	Time of intervention	Signal pathway	Main results	Author, reference
SD rats, CUMS	hippocampus, frontal cortex	GV20, EX-HN3, PC6	-* *-	QOD for 28 days	activation of BDNF pathway	elevation of BDNF mRNA and protein neural regeneration	Liang J, et al. 2012[104]

Male, SD rats, CUS	hippocampus	LI4, LR3	EA	QD for 21 days	regulation of soluble N-ethylmaleimide-sensitive factor attachment receptor proteins	depression of SNAP25, VAMP1, VAMP2, VAMP7, and syntaxin1	Fan L, et al. 2016[106]

SD rats, CUMS	hippocampus	GV20, EX-HN3	EA, 0.6mA, 2Hz	20min, QD for 21 days	activation of NO-cGMP pathway	elevation of nNOS, cGMP normalize activity of the NO/cGMP pathway	Han YJ, et al. 2009[107]

Male, SD rats, CUS	hippocampus	GV20, PC6	-* *-	QD for 28 days	Inactivation of NF-*κ*B inflammatory pathway	depression of NF-*κ*B, COX-2, prostaglandin inhibition of pro-inflammatory pathway	Shao RH, et al. 2015[108]

Male, SD rats, CUMS	hippocampus, prefrontal cortex	GV20, PC6	MA, rotated 2Hz for 1 min and retained	10min, QOD for 28 days	activation of ERK-CREB pathway	elevation of ratio of p-ERK1/2 to ERK1/2, ratio of p-CREB to CREB influence BDNF expression	Lu J, et al. 2013[109]

Male, SD rats, CUMS	hippocampus	GV20, GB34	EA, 0.3mA, 2/100Hz	30min, QD for 14 days	activation of ERK pathway	elevation of p-ERK neural stem cells proliferation	Yang L, et al. 2013[110]

Male, SD rats, CUMS	hippocampus	GV20, EX-HN3	EA, 1-3mA, 2Hz	15 min, QD for 14 days	modulation of the p-ERK1/2 and p-p38MAPK pathway	elevation of p-ERK1/2, p-p38	Xu J, et al. 2015[111]

Male, SD rats, CUMS	hippocampus	GV20, GV29	MA, 2Hz for 1min	10min, QD for 21 days	activation of ERK pathway	elevation of -ERK1/2, CREB, and p-CREB neurotrophy and neurogenesis	Zhang X, et al. 2016[112]

Male, SD rats, CUMS	hippocampus	GV20, GV29	EA, 0.6mA, 2Hz	20min, QD for 21 days	Activation of MAPK/ERK pathway	elevation of BDNF, ERK, pERK, ribosomal s6 kinase augmentation of BDNF pathway, neurogenesis, anti-apoptosis	Li W, et al. 2017[113]

Male, specific pathogen-free SD rats, CRS	hippocampus	GV20, GV29	EA, 1mA, 2Hz	pre-stress, 20min, QD for 28 days	modulation of MAPK/ERK pathway	elevation of MAPT depression of PKC inhibition of cell differentiation and proliferation	Yang X, et al. 2017[114]

Male, SD rats, CUMS	hippocampus	GV20, GV29	EA	21 days	inactivation of JNK pathway	depression of p-JNK anti-apoptosis	Dai W, et al. 2010[115]

Male, SD rats, CUMS	hippocampus	GV20, GV29	acupuncture	20 min, QD	inactivation of JNK pathway	depression of p-JNK protein, MKK 4, MKK 7 protein	Guo Y, et al. 2016[116]

Male, SD rats	hippocampus, serum	GV20, EX-HN1, ST36, ST40	EA	QOD for 21 days	regulation of hypothalamus-pituitary-adrenal axis	elevation of cortisol, PKA, PKC	Lu F, et al. 2008[117]

Male, SD rats, chronic mild stress	hippocampus	LI4, LR3	EA, 2/20 Hz	30min, QOD for 42 days	activation of AC-cAMP-PKA pathway	activation of AC-cAMP-PKA pathway	Liu JH, et al. 2012[118]

Male, SD rats, CUMS	hippocampus	GV20, EX-HN3	EA, 0.6mA, 2Hz	30min, QD for 14, 28 days	activation of CREB and BDNF pathways	elevation of BDNF, TrkB, PKA, pCREB depression of CaMKII anti-apoptosis, neuroprotection	Duan DM, et al. 2016[119]

Male, SD rats, CUMS	hippocampus	GV20, EX-HN3	MA,	pre-stress, 30min for 21 days	Activation of PKA/CREB pathway	elevation of PKA-*α* and p-CREB	Jiang H, et al. 2017[120]

Male, SD rats, Single prolonged stress	Hippocampus, serum	HT8	MA, rotate 2Hz for 30sec	QD	activation of mTOR pathway	elevation of corticosterone(serum), corticotropin-releasing factor, mTOR phosphorylation, Akt, ERK, p70S6K, p4E-BP-1, CREB, PSD95, Syn1, GluR1 increase synaptic plasticity	Oh JY, et al. 2018[121]

Male, Wistar rats, CUMS	hippocampus and serum	GV20, EX-HN3	EA, 1mA, 2Hz, pre-stress	60min, QD for 28 days	Regulation of neurotrophin signaling pathway, MAPK/ERK pathway and PI3K/Akt pathway	depression of miR-383-5p and miR-764-5p activation of neurotrophy and inhibition of abnormal apoptosis	Duan DM, et al. 2017[122]

Male, SD rats, CRS	hippocampus	GV20, EX-HN3	not mentioned	20min, QD for 28 days	down regulation of toll-like receptor signalling pathway and nucleotide-binding oligomerization domain-like receptor pathway	regulating inflammatory response, innate immunity and immune response	Wang Y, et al. 2017[123]

Male, SD rats, CRS	frontal cortex	GV20, GV29	MA	pre-stress, 20min, QD for 28 days	Toll-like receptor pathway, TNF pathway, NF-*κ*B pathway	inhibition of inflammatory process	Wang Y, et al. 2017[124]

Abbreviations

AC: adenyl cyclase; Akt: protein kinase B; BDNF: brain-derived neurotrophic factor; CaMK: Ca2+/calmodulin-dependent protein kinase; cAM: cyclic adenosine monophosphate; cGMP: cyclic guanosine monophosphate; COX: cyclooxygenase; CREB: phosphorylated cyclic AMP response element-binding protein; CRS: chronic restraint stress; CUMS: chronic unpredictable mild stress; CUS: chronic unpredictable stress; EA: electroacupuncture; ERK: extracellular signal-regulated kinase; JNK: c-Jun N-terminal kinases; MA: manual acupuncture; MAPK: mitogen-activated protein kinases; MAPT: microtubule-associated protein Tau; mRNA: messenger ribonucleic acid; mTOR: mammalian target of rapamycin; NF-*κ*B: nuclear factor kappa-light-chain-enhancer of activated B cells; nNOS: neuronal nitric oxide synthase; NO: nitric oxide; p38 MAPKs: p38 mitogen-activated protein kinases; PKA: protein kinase A; PKC: protein kinase C; QD: daily; QOD: every other day; SD rat: Sprague Dawley rat; TrkB: tyrosine receptor kinase B; VAMP: vesicle-associated membrane protein.

**Table 5:** Signal transduction pathways of acupuncture in treating Alzheimer's disease.

Subjects	Location	Acupoint	Intervention	Time of intervention	Signal pathway	Main results	Author, reference
Male, SD rat, scopolamine injection	brain	GV20	MA	pretreatment for 5 min, QD for 14 days	enhance cholinergic system-CREB-BDNF pathway	elevation of choline acetyltransferase, choline transporter 1, vesicular acetylcholine transporter, BDNF, CREB proteins neuroprotection	Lee B, et al. 2014[135]

APP/PS1 mice	brain	GV20	EA, 1/20 Hz	30min, QD for 4 weeks	modulation of BDNF-TrkB pathway	elevation of BDNF/proBDNF ratio, p-TrkB depression of *β*-amyloid (1-42), p75 anti-apoptosis	Lin R, et al. 2016[136]

Male, SAMP10	hippocampus	CV17, CV12, CV6, ST36, SP10	MA	QD	regulation of aging gene	elevation of p53, Mad related protein 2, Nucleoside diphosphate kinase B, AT motif-binding factor, Hsp84, Hsp86 depression of p38 MAPK, retinoblastoma-associated protein 1 anti-oxidation	Ding X, et al. 2006[18]

SD rat, A*β*1-40 injection	hippocampus, frontal cortex	GV20, KI3, ST36	EA, 1mA, 2Hz	15min, QD for 12 days	inactivation of p38 MAPK pathway	depression of p-p38 MAPK protein, IL-1beta mRNA decrease neuroinflammation	Fang JQ, et al. 2013[137]

Male, SD rat, A*β*1-40 injection	hippocampus CA1	GV20, BL23	EA, 2mA, 2-4V, 2Hz	20min, QD, 6 days/ wk for 4 weeks	activation of PPAR-*γ* pathway	elevation of PPAR-*γ* depression of p-p38MAPK, A*β*, p-Tau Ser404 protein decrease neuroinflammation	Zhang M, et al. 2017[138]

SAMP 10 mice	neocortex and hippocampus	CV17, CV12, CV6, SP10, ST36	not mentioned	QD for 14 days	p 130 pathway	elevation of p130 cell proliferation	Liu T, et al. 2008[139]

Male, SAMP8 mice	cortex and hippocampus	CV17, CV12, CV6, ST36, SP10	MA, >2Hz	30sec per acupoint, QD, 21 days	regulation of G protein/ IP3/ Ca2+ amplitude pathway	elevation of physiologically coupled activation rate and maximal coupled activation rate of G*α*s and G*α*i signal homeostasis	Luo B, et al. 2017[140]

Male, APP/PS1 mice	brain	GV20	EA, 1mA, 2/15Hz	30min, QD, 5 days/wk for 4 weeks	suppression of astrocytic NDRG2 pathway	depression of Glial fibrillary acidic protein, NDRG2 increase astrocytic reactivity	Wang F, et al. 2014[141]

telomerase-deficient mice(TERC-/-) mice	hippocampus and dentate gyrus	ST36	MA	30 min, QD for 4 days	activation of BDNF pathway	elevation of BDNF, TrkB, p75NTR, Akt, and ERK1/2 increase telomerase activity	Lin D, et al. 2015[142]

SD rat, beta-amyloid (Abeta)(1-40) injection	hippocampal	LI20, EX-HN3	EA, 1-3mA, 80-100Hz	10min, QD, 5 days/wk for 6 weeks	regulation of Bcl-2/Bax	elevation of Bcl-2 depression of Bax anti-apoptosis	Liu ZB, et al. 2011[143]

Male, SD rat, A*β*1-40 injection	hippocampus CA1	GV20, BL23	EA, <2mA, 20Hz	30 min, QD, 6 days/ wk for 4 weeks	downregulation of Notch pathway	elevation of Bcl-2, synapsin-1, synaptophysin depression of Bax, Notch1 mRNA, Hes1 mRNA anti-apoptosis	Guo HD, et al. 2015[144]

Male, APP/PS1 mice	hippocampus	GV20, KI1	EA, 1mA, 2/100Hz	15min, QD for 3 days	inactivation of caspase-3/ Bax pathway	elevation of Bcl-2/Bax ratio depression of caspase-3-positive cell number and Bax protein anti-apoptosis	Li XY, et al. 2016[145]

APP/PS1 mice	hippocampus, cortex	GV20	EA, 1/20Hz	30min, QD, 5 days/wk for 4 weeks	regulation of AMPK/mTOR pathway	elevation of GLUT1, GLUT3, p-AMPK, p-Akt, mTOR decrease A*β* (1-42) deposition, decrease autophagy process	Liu W, et al. 2017[148]

Male, SAMP8 mice	hippocampus CA1	GV14, BL23	EA, 1mA, 2Hz	20min, QD, 8 days' treatment and 2 days' rest for 3 cycles	activation of AMPK pathway	elevation of p-AMPK balance energy metabolism and improved cognitive impairment	Dong W, et al. 2015[20]

Male, SAMP8 mice	hippocampus and frontal cortex	GV14, BL23	EA, 1mA, 2Hz	20min, QD, 8 days' treatment and 2 days' rest for 3 cycles	activation of SIRT1-dependent PGC-1*α* expression pathway	elevation of ATP levels, SIRT1, PGC-1*α* depression of PGC-1*α* acetylation improved brain energy metabolism	Dong W, et al. 2015[151]

Abbreviations

Akt: protein kinase B; AMPK: AMP-activated protein kinase; APP/PS1: amyloid precursor protein (APP)/presenilin-1 (PS1) double transgenic; Bax: Bcl-2 associated X; Bcl-2: B-cell lymphoma 2; BDNF: brain-derived neurotrophic factor; CREB: phosphorylated cyclic AMP response element-binding protein; EA: electroacupuncture; ERK: extracellular signal-regulated kinase; GLUT: glucose transporter; IL: interleukin; IP3: Inositol triphosphate; MA: manual acupuncture; MAPK: mitogen-activated protein kinases; NDRG2: N-myc downregulated gene 2; NMDA: N-methyl-D-aspartate; PGC1: proliferator-activated receptor *γ* coactivator 1; PPAR-*γ*: peroxisome proliferator-activated receptors *γ*; QD: daily; QOD: every other day; RBL2: Retinoblastoma-like protein 2; SAMP: senescence-accelerated mouse prone; SD rat: Sprague Dawley rat; SIRT1: sirtuin 1; TrkB: tyrosine receptor kinase B.

**Table 6:** Signal transduction pathways of acupuncture in treating vascular dementia.

Subjects	Location	Acupoint	Intervention	Time of intervention	Signal pathway	Main results	Author, reference
Male, Wistar rats, homologous blood emboli injection of internal carotid artery	hippocampus	ST36	MA	QD for 14 days	activation of cAMP/PKA/CREB pathway	activation of long-term potentiation	Li QQ, et al. 2015[47]

Male, SD rats, MCAO	hippocampus	GV24, GV20	EA, 1-3mA, 5/20Hz	30min, QD	increase expression of p-CREB	elevation of superoxide dismutase and glutathione peroxidase, Bcl-2 depression of malondialdehyde, Bcl2-xl anti-oxidase and anti-apoptosis	Lin R, et al. 2015[48]

Female, SD rats, MCAO	hippocampus	GV20, GV24	EA, 1/20Hz	30min, QD for 7 days	inactivation of CaM-CaMKIV-CREB pathway	anti-apoptosis	Zhang Y, et al. 2016[50]

Male, SD rats, MCAO	hippocampus	GV20, HT7	MA, LA, 30 mW, 100Hz	14 days	enhance cholinergic system	elevation of CREB, BDNF and Bcl-2 depression of Bax anti-apoptosis	Yun YC, et al. 2017[51]

Mongolian gerbils, CCAO	hippocampal CA1	KI3, GV20	EA, 1mA, 2Hz	20 min, 4 times/ 2 days	regulate MAPK/ERK pathway	elevation of p-ERK depression of ionized calcium-binding adaptor molecule 1, TLR4, TNF-*α* decrease neuroinflammation, regulate the synaptic plasticity	Yang EJ, et al. 2016[153]

Male Wistar rats, two-vessel occlusion model	hippocampus	GV20, ST36	MA	QD for 14 days	inactivation of ASK1-JNK/p38 pathway	elevation of thioredoxin-1 and thioredoxin reductase-1 anti-oxidase and anti-apoptosis	Zhu W, et al. 2018[154]

Male, Wistar rat, homoblood injection	hippocampal CA1	CV17, CV12, CV6, ST36, SP10	MA, 2Hz	30sec for each acupoint, QD, 6 days/ wk for 3 weeks	balance Bcl-2 and Bax expression	elevation of Bcl-2 depression of Bax anti-apoptosis	Wang T, et al. 2009[155]

Male, SD rat, using modified Pulsinelli 4-vessel-occlusion method	hippocampal CA1	Scalp-acupuncture	MA	30min, QD for 10 days	activation of Bcl-2 pathway	elevation of Bcl-2 anti-apoptosis of astrocytes	Tian WJ, et al. 2015[156]

Female, SD rat, CCAO	hippocampus	GV20, GV14, BL23	EA, 2mA, 4Hz	30min, QD for 30 days	activation of mTOR pathway	elevation of mTOR and eIF4E modulates cell growth, proliferation and synaptic plasticity	Zhu Y, et al. 2013[157]

Abbreviations

ASK1: apoptosis signal-regulating kinase 1; Bax: Bcl-2 associated X; Bcl-2: B-cell lymphoma 2; BDNF: brain-derived neurotrophic factor; CaMK: Ca2+/calmodulin-dependent protein kinase; cAMP: cyclic adenosine monophosphate; CCAO: occlusion of common carotid artery; CREB: phosphorylated cyclic AMP response element-binding protein; EA: electroacupuncture; eIF4E: eukaryotic translation initiation factor 4E; ERK: extracellular signal-regulated kinase; JNK: c-Jun N-terminal kinases; MA: manual acupuncture; MAPK: mitogen-activated protein kinases; MCAO: occlusion of middle cerebral artery; mTOR: mammalian target of rapamycin; PKA: protein kinase A; QD: daily; TLR4: Toll-like receptor 4.

**Table 7:** Signal transduction pathways of acupuncture in treating Parkinson's disease.

Subjects	Location	Acupoint	Intervention	Time of intervention	Signal pathway	Main results	Author, reference
Male SD rats, rotenone injection	substantia nigra	GV16, LR3	EA, 1mA, 2Hz	20min, QD for 14 days	inactivation of p38-MAPK pathway	elevation of tyrosine hydroxylase-positive neuron depression of phosphorylated p38-MAPK, COX-2 decrease neuroinflammation	Wang SJ, et al. 2013[158]

Male SD rats, rotenone injection	substantia nigra	GV16, LR3	EA, 2mA, 2Hz	20min, QD for 14 days	inactivation of ERK 1/2 pathway	elevation of tyrosine hydroxylase protein depression of p-ERK 1/2, TNF-*α*, IL-1*β* decrease neuroinflammation	Wang SJ, et al. 2014[159]

Male C57BL/6 mice, MPTP injection	substantia nigra, striatum	GB34	MA, 2Hz for 15sec	QD for 7 days	activation of PI3K/Akt pathway	elevation of pAkt prevents MPTP-induced dopaminergic neuron degeneration	Kim SN, et al. 2011[160]

Male C57BL/6 mice, MPTP injection	substantia nigra pars compacta, striatum	GB34	MA, 2Hz for 15sec	QD for 12 days	activation of PI3K/Akt pathway	elevation of dopamine depression of dopamine- and cAMP-regulated phosphoprotein of 32 kDa, Fos increase dopamine turnover rate	Kim SN, et al. 2011[161]

Male, C57BL6 mice (MPTP intraperitoneal injection) and SD rats (Sigma-Aldrich injection into substantia nigra)	substantia nigra	GB34, LR3	EA, 1mA, 50Hz	QD for 5(mice)/7(rats) days	activation of Akt pathway	elevation of BDNF, Bcl-2, tyrosine hydroxylase regulation of cell cycle	Lin JG, et al. 2017[162]

Imprinting control region mouse pups, systemic 6- hydroxydopamine injection	hippocampus	KI3	EA, 1mA, 2Hz	15min, QD, 5 days/wk for 6wks	inactivation of pPKA/pPKC/CaMKII*α* signaling pathways	depression of pNR1, pNR2B, pPKA, pPKC, pCaMKII*α*, pERK, pCREB reduce neuronal excitotoxicity	Lu KW, et al. 2017[163]

Male C57BL/6 mice, MPTP injection	substantia nigra par compacta	GB34	MA, 2Hz for 15sec every 5min	10min, QD for 7 days	m-TOR independent pathway	depression of *α*-synuclein induces autophagic clearance of *α*-syn, dopaminergic neurons protection	Tian T, et al. 2016[164]

Male C57BL/6 mice, MPTP injection	substantia nigra, striatum	GB34, GB39	EA, 1mA, 100Hz	20min, QD for 12 days	regulation of glyoxalase system	elevation of tyrosine hydroxylase-positive neurons, cytochrome c oxidase subunit Vb depression of cytosolic malate dehydrogenase, munc18-1, hydroxyacylglutathione hydrolase anti-oxidative effect	Kim ST, et al. 2010[165]

Male C57BL/6 mice, MPTP injection	midbrain, striatum	ST36, SP6	EA, 1-1.4mA, 100Hz	30min, QD for 12 days, except day 7	activation of Nrf2-ARE pathway	elevation of tyrosine hydroxylase, ARE-driven reporter gene, NQO1, HO-1 depression of ionized calcium-binding adaptor molecule 1, TNF-*α*, IL-6, IL-1*β* anti-oxidative effect	Lv E, et al. 2015[166]

GFAP-tTA/tetO-*α*-syn double transgenic mice	midbrain, striatum	ST36, SP6	EA, 1-1.2mA, 100Hz	30min, QD for 28 days	activation of Nrf2-ARE pathway	elevation of Nrf2, HO-1, glutamate-cysteine ligase modifier subunits depression of *α*-syn decrease astrogliosis and neuroinflammation	Deng J, et al. 2015[167]

Male C57BL/6 mice, MPTP injection	striatum, substantia nigra	GB34	MA, 2Hz, 15sec	QD for 12 days	activation of p53 signaling pathways	elevation of p53 dopaminergic neuron protection	Park JY, et al. 2015[168]

Abbreviations

Akt: protein kinase B; ARE: antioxidant response element; CaMK: Ca2+/calmodulin-dependent protein kinase; cAMP: cyclic adenosine monophosphate; COX: cyclooxygenase; CREB: phosphorylated cyclic AMP response element-binding protein; EA: electroacupuncture; ERK: extracellular signal-regulated kinase; HO-1: heme oxygenase-1; IL: interleukin; MA: manual acupuncture; MPTP: 1-methyl-4-phenyl-1,2,3,6-tetrahydropyridine; mTOR: mammalian target of rapamycin; NQO1: nicotinamide adenine dinucleotide phosphate quinone oxidoreductase; Nrf2: nuclear factor erythroid 2-related factor 2; p38 MAPKs: p38 mitogen-activated protein kinases; PI3K: phosphatidylinositol-4,5-bisphosphate 3-kinase; PKA: protein kinase A; PKC: protein kinase C; pNR: phosphorylated N-methyl-D-aspartate receptor; QD: daily; SD rat: Sprague Dawley rat; TNF-*α*: tumor necrosis factor-alpha.

## Data Availability

The data in this study are available to other researchers upon request.
